# Using Time Perception to Explore Implicit Sensitivity to Emotional Stimuli in Autism Spectrum Disorder

**DOI:** 10.1007/s10803-017-3120-6

**Published:** 2017-04-20

**Authors:** Catherine R. G. Jones, Anna Lambrechts, Sebastian B. Gaigg

**Affiliations:** 1 School of Psychology, Cardiff University, Tower Building, Cardiff, CF10 3AT UK; 2 Department of Psychology, City University London, Northampton Square, London, EC1V 0HB UK

**Keywords:** Autism, Emotion, Face processing, Temporal bisection, Time perception, Arousal

## Abstract

Establishing whether implicit responses to emotional cues are intact in autism spectrum disorder (ASD) is fundamental to ascertaining why their emotional understanding is compromised. We used a temporal bisection task to assess for responsiveness to face and wildlife images that varied in emotional salience. There were no significant differences between an adult ASD and comparison group, with both showing implicit overestimation of emotional stimuli. Further, there was no correlation between overestimation of emotional stimuli and autistic traits in undergraduate students. These data do not suggest a fundamental insensitivity to the arousing content of emotional images in ASD, or in individuals with a high degree of autistic traits. The findings have implications for understanding how emotional stimuli are processed in ASD.

## Introduction

Difficulties in understanding and responding appropriately during social exchange are hallmarks of autism spectrum disorder (ASD). These difficulties have led to close scrutiny of the ability to process emotional cues, with a heavy emphasis on recognising emotion in the face (Uljarevic and Hamilton 2013). Investigation of facial emotion recognition in ASD has typically involved labelling faces expressing the six basic emotions (happiness, sadness, fear, anger, surprise, disgust). Against a background of mixed findings, recent meta-analyses have concluded that difficulties in facial emotion recognition are characteristic of ASD, although the severity of impairment varies according to emotion (Lozier et al. 2014; Uljarevic and Hamilton 2013). However, a challenge of any task that involves participants explicitly engaging with the process being measured is that they may use alternative strategies to ‘hack out’ the correct response. For instance, similar behavioural emotion recognition performance in participants with and without ASD is found alongside different patterns of neural activation (e.g. Rahko et al. 2012). A related issue is that uninterrupted time to decide a person’s emotional state does not recreate the demands of real-life social interactions. Therefore, it is arguable that explicit and relatively straightforward measures of emotion recognition provide only limited insight into the more complex realities of processing emotion in ASD.

**Fig. 1:**
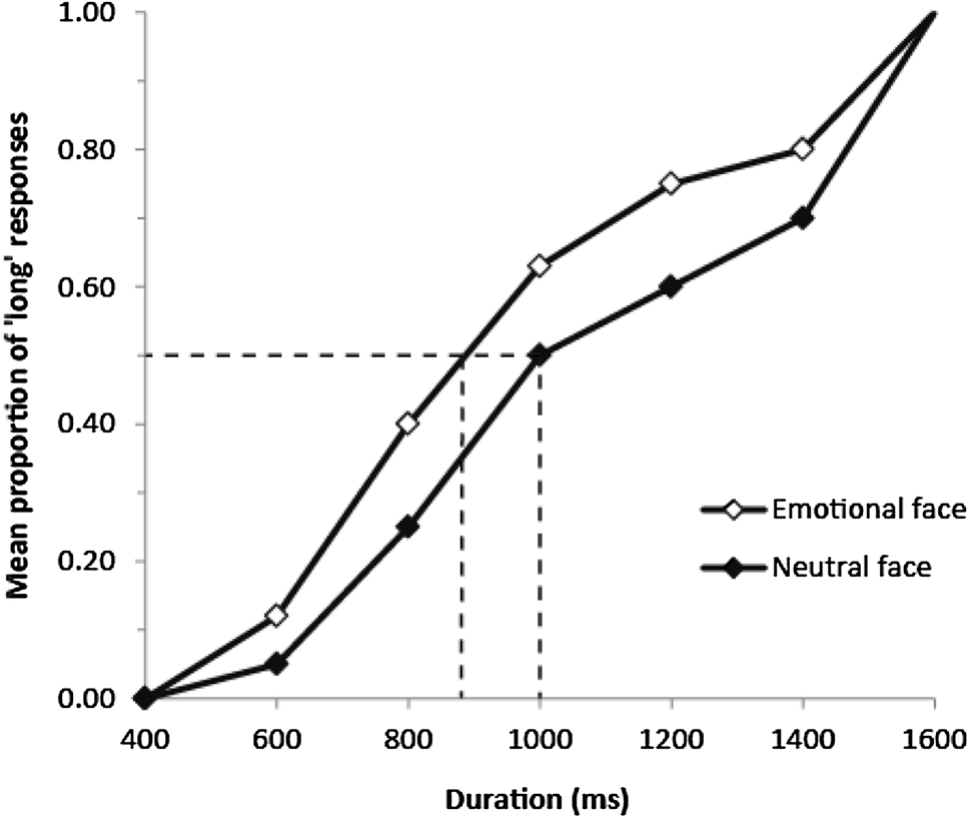
Hypothetical psychophysical functions to illustrate the effect of emotional stimuli on duration judgements in the temporal bisection task. The mean proportion of long responses is plotted against stimulus duration. The leftward shift of the emotional face data along the x axis illustrates the response bias towards long responses. *Dashed lines* illustrate the bisection point (x axis), which is the plotted duration where 50% of responses are long (y axis) (see Method for further explanation)

One way of circumventing these issues is to measure emotion processing indirectly. For example, in circumstances where implicit processing of the emotional content of stimuli will influence the response, despite no explicit instruction to pay attention to emotion. A similar approach has been taken in characterising theory of mind in ASD, with explicit mentalising being ostensibly unimpaired while implicit and intuitive mentalising abilities are compromised (Senju et al. 2009). For emotion processing, an elegant paradigm that achieves this goal is an adapted version of the temporal bisection task (Droit-Volet et al. 2004). The temporal bisection task is a classic measure of interval timing that was originally used in animals (Wearden 1991). Within the last 25 years, the task has helped characterise the mechanistic structure and psychophysical hallmarks of human perceptual timing in the millisecond- and seconds-range (see Jones and Jahanshahi 2014 for a summary of related tasks). It has been argued that a brain-based internal clock (or clocks) govern this distinct type of timing process (see Buhusi and Meck 2005). The temporal bisection task requires participants to learn short and long standard durations (e.g. 400 and 1600 ms), typically presented as simple visual displays. During the testing phase, stimuli are presented for both the standard and intermediate durations and participants have to classify each as more similar to the short or long standards. The proportion of ‘long’ responses increases monotonically with stimulus duration and can be plotted as a psychophysical function, or bisection curve, with duration along the x axis. Various performance measures can be obtained from the psychophysical function, with the steepness of the slope indexing temporal sensitivity and the lateral displacement along the x axis indexing response bias (see Wearden 1991). In typical populations, when face stimuli are used during the testing phase the duration of emotional faces are consistently overestimated compared to neutral faces, which is demonstrated in the leftward displacement of the bisection curve (e.g. Droit-Volet et al. 2004; Effron et al. 2006; Fayolle and Droit-Volet 2014; Tipples 2008, 2011; Tipples et al. 2015). In contrast to response bias, temporal sensitivity is typically not affected (see Fig. 1 for a hypothetical illustration of typical findings). The intuitive explanation is that implicit recognition of the emotional content of the stimuli is driving the effect.

One explanation for the findings is that the internal clock that times the intervals is sensitive to the arousal induced by viewing emotional faces (see Cheng et al. 2016; Droit-Volet and Meck 2007). In essence, the internal clock is speeded by the increased levels of arousal, which means that more clock ‘ticks’ (temporal units) are accrued and the period of time is judge as longer. This explanation has been interpreted within the most common internal clock model, scalar expectancy theory (SET; Gibbon 1977; Gibbon et al. 1984). SET is an information processing model that conceives that time is processed within a clock system consisting of a pacemaker that emits pulses, which are passed via a switch to an accumulator that represents current elapsed time. Working memory and reference memory processes are used to store time values and a comparator, or decision making process, compares these values to enable a temporal judgement to be made. Despite criticisms of SET (e.g. Buhusi and Meck 2005; Droit-Volet and Meck 2007), the explanation of overestimation being driven by a speeded pacemaker and increased accumulation of temporal units aligns with evidence demonstrating that stimulant drugs lead to overestimation of duration (see Coull et al. 2011; Droit-Volet et al. 2013). This arousal-based explanation also fits the subjective phenomenon of time seeming to slow when in a highly arousing situation such as an accident. A range of evidence has indicated that emotional stimuli trigger activation of the sympathetic autonomic nervous system (e.g. Brouwer et al. 2013). Direct physiological evidence for increased arousal during the emotional temporal bisection task has remained unexplored, partly as the multiple short trials do not lend themselves to accommodating the refractory periods of physiological responses. However, Gil and Droit-Volet (2012) found that emotional images that were subjectively judged as highly arousing produced greater overestimation than images with lower ratings. Mella et al. (2011) directly measured skin conductance response (SCR) during a duration and emotion discrimination paradigm. High arousing sounds were judged as longer and led to enhanced SCR when participants attended to the emotional intensity of the stimuli, although not all data were compatible with a simple relationship between time, arousal and emotion. Regardless of the physiological underpinnings, overestimation of emotional stimuli is a reliable finding that can be used as an indirect index that the emotional salience of the stimuli has been processed.

The emotional temporal bisection task can therefore give insight into whether the implicit response to emotion is intact in ASD, which is important for illuminating the precise nature of the emotional processing difficulties experienced in ASD. As performance requires a timing judgement, the task also provides information on the accuracy of perceptual timing in ASD. Previous research has argued that there is a fundamental timing difficulty in ASD (Allman et al. 2011; Brodeur et al. 2014; Falter et al. 2012; Karaminis et al. 2016; Kargas et al. 2015; Maister and Plaisted-Grant 2011; Martin et al. 2010; Szelag et al. 2004). However, this is not a universal finding (Jones et al. 2009; Gil et al. 2012; Mostofsky et al. 2000; Wallace and Happé 2008) and the debate remains open. An important secondary aim of the study, therefore, is to add to the small but growing body of literature that considers whether interval timing poses difficulties for individuals with ASD.

As well as investigating the implicit responses to emotional faces in ASD, the studies reported below also examined responses to a set of wildlife images (e.g., spider) that were chosen to vary in emotional salience to a similar extent as the face stimuli. Emotion research in ASD often focuses on the human face, making it difficult to determine whether observed effects are face-specific or reflect a more general difficulty with emotion processing (see Gaigg 2012). Our study was piloted in a large population of typically developing (TD) young adults, reported in Study 1, in which the Autism Quotient (AQ: Baron-Cohen et al. 2001) was used to investigate if there was any meaningful association between task performance and self-reported autistic traits in the general population. Study 2 directly compared adults with ASD to a comparison group without a diagnosis. A reduced emotional temporal bisection effect in ASD would suggest atypical implicit responsiveness to emotional stimuli, whereas an intact emotional temporal bisection effect would indicate that this response, thought to be mediated by sub-cortical arousal mechanisms, is functioning typically.

## Study 1: Temporal Bisection of Arousing Face and Wildlife Images in a Typical Adult Population

### Method

#### Participants

Eighty-five undergraduate and postgraduate students (47 female; *M* = 22 years 7 months; SD = 4.94) from the University of Essex participated. There was no significant difference in age between male and female participants (see Table 1). None of the participants had a history of psychiatric or neurological disorder or illness. As one of the tasks included pictures of spiders, all participants were screened for arachnophobia. No participant had a diagnosis of ASD, or a family member with ASD. An additional four participants were tested but their data in both the face and wildlife conditions were discarded because their responses across the varying durations of the stimuli did not conform to a sigmoid curve (see below), which suggests that they did not follow the task instruction (i.e., the participants did not discriminate between shorter and longer durations). All participants gave informed consent and the study was approved by the Ethics Committee of the University of Essex.

**Table 1:** Summary of participants in Study 1

Measure	Females (n = 47)		Males (n = 38)		Total (n = 85)	
	*M*	*SD*	*M*	*SD*	*M*	*SD*
Age (years; months)	22;0	3.00	23;5	6.57	22;7	4.94
AQ^a^	14.09	5.83	16.11	5.00	15.0	5.51

^a^Autism Spectrum Quotient (Baron-Cohen et al. 2001)

### Materials and Procedure

#### Temporal Bisection Tasks

The tasks were programmed in E-Prime 2.0 (Schneider et al. 2002) and displayed on a PC. The experiment consisted of two versions of a temporal bisection task in which participants first learned to discriminate between a short and a long reference duration and then tried to categorise varying durations as more similar to the short or long exemplars. The training phase of each version consisted of 20 trials in which a monochrome grey rectangle (15 cm × 19.3 cm) appeared for either 400 or 1600 ms on a computer monitor. These durations served as the short and long reference durations. During the first 10 training trials the duration of the rectangle alternated, accompanied by a visual display of the appropriate label (‘short’ or ‘long’). For the final 10 training trials, the rectangle appeared randomly for either 400 or 1600 ms and participants were required to label the trial as either short or long by pressing appropriate response keys following the on-screen question, ‘Do you think this was SHORT or LONG?’. Throughout the task, the ‘N’ key (re-labelled ‘S’) on the computer keypad was used for a short response and the ‘M’ key (re-labelled ‘L’) was used for a long response. Participants used their preferred hand/fingers to respond.

Two versions of the bisection task were administered in counterbalanced order across participants. In the face version, photographs of four Caucasian male models (#23, 26, 27 and 36) were selected from the NimStim database (Tottenham et al. 2009), with each posing neutral, happy, angry and fearful expressions (i.e. 16 face stimuli). In the wildlife version, photographs of four different flowers, puppies, snarling canine/felines (snarl) and spiders were sourced from various web-sites (i.e. 16 wildlife stimuli). The wildlife stimuli were chosen because they were hedonically similar to the neutral, happy, angry and fearful facial expressions, respectively.

All experimental stimuli were converted to 24-bit grey-scale images and cropped to match the dimensions of the grey rectangle used for training. In each of the two versions of the task, the 16 stimuli (4 per hedonic category) were presented once each at 7 different durations (400, 600, 800, 1000, 1200, 1400 and 1600 ms) for a total of 112 trials. The order of presentation was pseudo-randomised with the constraint that no more than 2 successive trials could be of the same duration or hedonic category. Each trial began with a ‘READY’ screen that lasted randomly between 1800 and 2500 ms and was followed by the experimental stimulus at one of the pre-set durations. A blank interval lasting between 200 and 500 ms separated the stimulus from the response prompt, ‘Do you think this was SHORT or LONG’?’. The prompt terminated with the participants’ response and was followed by another 200–500ms blank interval before the ‘READY’ signal reappeared to mark the beginning of the next trial.

To establish that the images produced the predicted subjective feelings of arousal, the participants were required to rate each image for valence and arousal using the Self-Assessment Manikin (SAM: Lang 1980). The SAM uses cartoon images to represent 9-point scales of arousal and valence. The images (16 face and 16 wildlife) were presented in a random order and at a self-paced rate immediately after completion of the temporal bisection tasks. Fifty-four participants completed this stage of the study.

The AQ questionnaire (Baron-Cohen et al. 2001) was used to measure self-reported autistic traits (range available = 0–50). This was administered at the end of the testing session.

#### Analysis of the Temporal Bisection Data

The proportion of long responses (p(long)) for each category and stimulus duration was calculated (i.e. proportion of long responses out of 4). In addition, we fitted participants’ response to a cumulative Gaussian sigmoid using the psignifit MATLAB Toolbox (Wichmann and Hill 2001a, b) and extracted the bisection points of the resulting response curves for each individual. The bisection point is the point of subjective equality, i.e. the duration at which short and long responses occur with equal probability. It reflects accuracy in relation to the veridical middle point, with lateral displacement indicating response bias towards either short or long responses. It can be measured as the x-axis value at which sigmoid functions cross the 50% midpoint of the y-axis (p(long) = 0.5). For the current experiment this would be expected to be close to 1000 ms (i.e. half way between the shortest and longest durations). Following similar principles the Weber ratio can be calculated, which reflects the slope of the sigmoid curve and serves as an index of temporal sensitivity. It is half the difference between the upper difference limen (p(long) = 0.75) and the lower difference limen (p(long) = 0.25) divided by the bisection point. A lower score indicates greater temporal sensitivity, reflected in a steeper slope. As the p(long) and bisection point data both enable inspection of response bias and to reduce the amount of analysis reported, our main analysis focuses on the p(long) and Weber ratio. The bisection data are provided in tables for interest, presented alongside the Weber ratio data, and are the index of temporal overestimation that are correlated with the AQ.

To identify participants who may not have followed the instructions and therefore performed no better than chance, we applied a best-fit computation to compare the quality of the response curve within two different models. Using MATLAB, the response curve produced for each participant for each stimulus type was analysed to establish if a sigmoid curve (two-parameter fit) or a horizontal line (one-parameter fit, indicting no differentiation in performance by image duration) was a significantly better fit. If a sigmoid function did not best-fit a participant’s data for any one of the images in a given condition (face or wildlife) then the participant was excluded from that condition.

Analysis of the data was conducted in SPSS (IBM Corp, Version 20.0) using repeated measures analysis of variance (ANOVA). Hypothesis significance testing was supplemented by the calculation of effect size as well as the 90% confidence intervals for the effect sizes (Lakens 2013; Steiger 2004). Effect sizes were calculated using partial eta squared (ƞ^2^_p_), where a small effect is considered 0.01, a medium effect 0.06 and a large effect 0.14.

## Results

One participant’s face data was lost for technical reasons and another participant did not produce a best-fit sigmoid-shaped curve (therefore, face n = 83). For the wildlife images, one participant did not complete the task due to self-reported arachnophobia and a further three participants did not produce a best-fit sigmoid-shaped curve (therefore, wildlife n = 81).

### Autism Spectrum Quotient

Scores ranged from 2 to 29, with no individuals scoring above the cut-off (32) for clinical significance (Baron-Cohen et al. 2001). The mean score for the group was 15.0 (SD = 5.5) with a median of 15.0. There was no significant difference between the scores for males and females (see Table 1).

### Subjective Valence and Arousal Ratings for the Face and Wildlife Data

Using two repeated measures ANOVAs to separately examine the face and wildlife data (see Table 2), both showed a main effect of emotion on the arousal ratings (Face: F (3, 159) = 15.29; p < .001, ƞ^2^_p_=0.22, 90% CI [.13,.30]; Wildlife: F (3, 159) = 17.86; p < .001, ƞ^2^_p_=0.25, 90% CI [.15, .33]). Planned simple contrasts indicated emotional faces were rated as significantly more arousing than the neutral face (all p < .001, ƞ^2^_p_ range 0.24–0.38) and emotional wildlife images were significantly more arousing than the neutral flower (all p < .001, ƞ^2^_p_ range 0.32–0.41). For the valence ratings (see Table 2), repeated measures ANOVAs again showed main effects of emotion (Face: F (3, 159) = 69.80; p < .001, ƞ^2^_p_=0.57, 90%CI [.48, .63]; Wildlife: F (3, 159) = 114.20; p < .001, ƞ^2^_p_=0.68, 90% CI [.61,.73]). For the face stimuli, neutral faces were rated as more positive than angry and fearful faces and less positive than happy faces (all p < .01, ƞ^2^_p_ range 0.11–0.69). Similarly, for the wildlife images, flower images were rated as more positive than spider and snarl images but less positive than puppy images (all p < .001, ƞ^2^_p_ range 0.28–0.66).

**Table 2:** Average valence and arousal ratings for the face and wildlife stimuli. Score range was 1–9, with a high score indicating the image produced more positive valence and greater arousal

	*Valence*		*Arousal*	
	*M*	*SD*	*M*	*SD*
*Face*				
Angry	3.90	1.60	3.73	1.98
Fear	4.41	1.54	3.88	1.89
Happy	7.16	1.33	3.40	2.07
Neutral	4.99	0.68	2.45	1.48
*Wildlife*				
Snarl	3.67	1.36	4.42	1.94
Spider	2.99	1.72	5.02	2.52
Puppy	7.51	1.39	3.79	2.37
Flower	6.50	1.41	2.38	1.81

### Temporal Bisection: Face

**Fig. 2:**
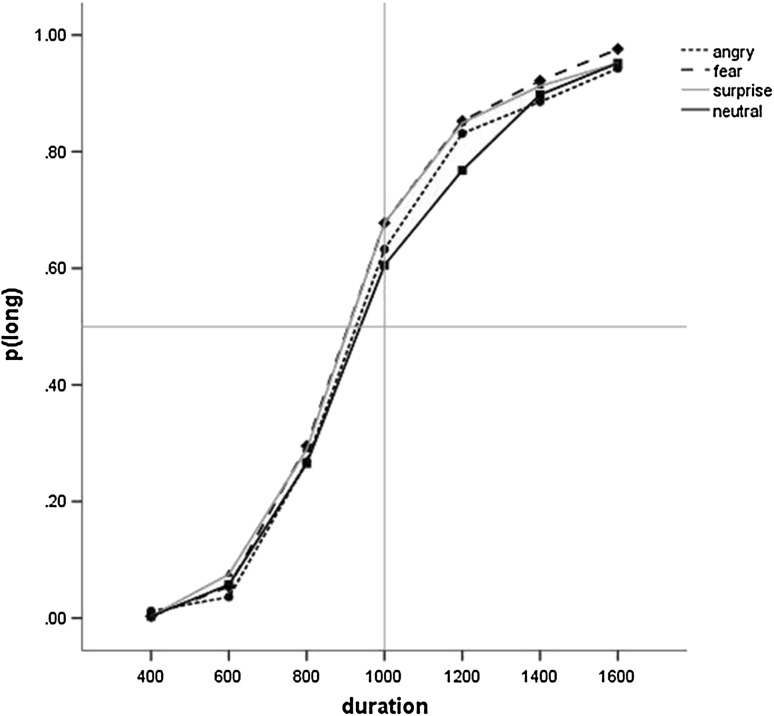
Proportion of long responses for the four facial expressions in Study 1. For clarity, error bars are omitted

A 4(Emotion) × 7(Duration) ANOVA of p(long) showed a main effect of Duration (F(3.04, 249.03) = 995.83, p < .001, ƞ^2^_p_=0.92, 90% CI [.91, .93]), confirming that long responses increased with stimulus duration, and Emotion (F(3, 246) = 6.16, p < .001, ƞ^2^_p_=0.07, 90% CI [.02, .12]) (see Fig. 2). Planned simple contrasts indicated that fearful (F(1, 82) = 13.70, p = .001, ƞ^2^_p_=0.14, 90% CI [.05, .26]) and happy (F(1, 82) = 13.05, p = .001, ƞ^2^_p_=0.14, 90% CI [.04, .25]) faces elicited a significantly higher proportion of long responses than neutral faces. There was no difference between angry and neutral faces (p > .3, ƞ^2^_p_=0.01, 90% CI [.00, .07]). The interaction of Emotion and Duration was not significant (p > .2, ƞ^2^_p_=0.01, 90% CI [.00, .02]).

Parallel analyses of the Weber ratio (see Table 3) did not produce significant findings (p > .7; ƞ^2^_p_=0.006, 90% CI [.00, .02]) indicating that accuracy but not sensitivity was affected by emotional faces.

**Table 3:** Average bisection points (ms) and Weber ratio for all stimulus categories for the face and wildlife images in Study 1

	*Bisection point*		*Weber ratio*	
	*M*	*SD*	*M*	*SD*
*Face*				
Angry	967.93	155.95	0.116	0.08
Fear	944.97	142.73	0.123	0.09
Happy	941.66	150.34	0.120	0.08
Neutral	982.82	177.04	0.131	0.13
*Wildlife*				
Snarl	945.65	174.61	0.101	0.07
Spider	976.12	157.91	0.122	0.09
Puppy	963.90	150.05	0.108	0.08
Flower	997.29	155.34	0.123	0.08

### Temporal Bisection: Wildlife

**Fig. 3:**
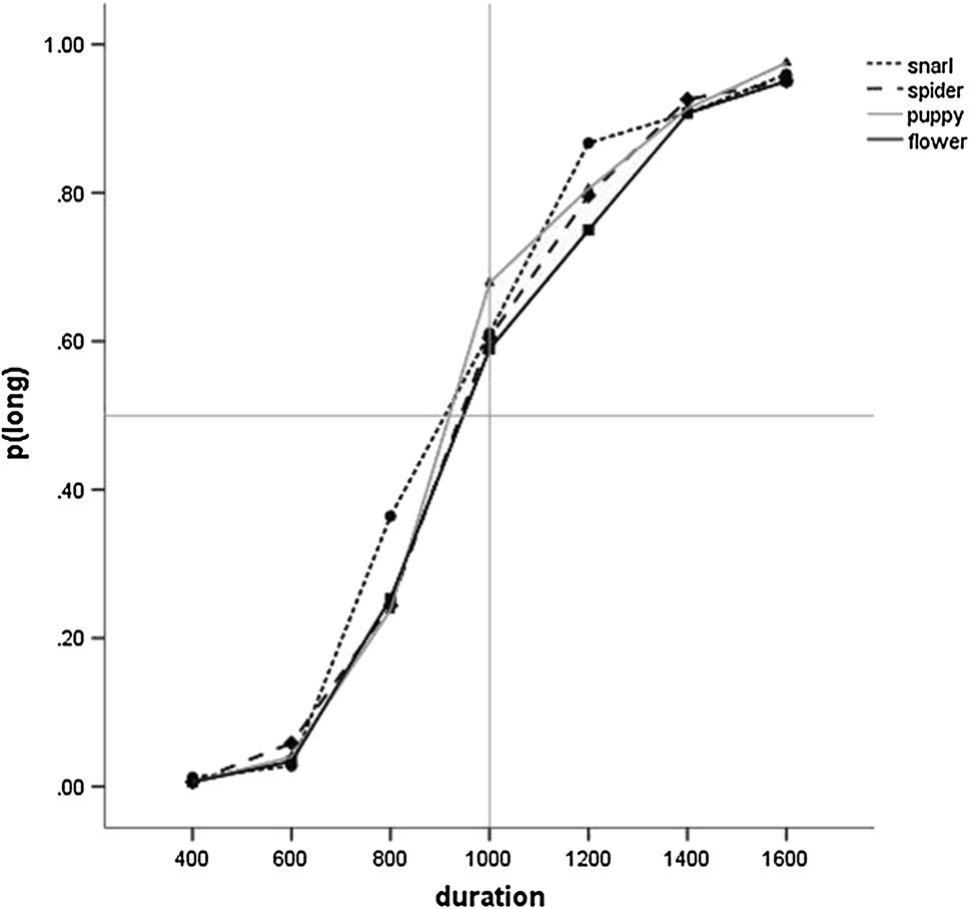
Proportion of long responses for the four wildlife images in Study 1. For clarity, error bars are omitted

A 4(Emotion) × 7(Duration) ANOVA of p(long) showed a main effect of Duration (F(2.86, 228.79) = 958.65, p < .001, ƞ^2^_p_=0.92, 90% CI [.91, .93]) and Emotion (F(3, 240) = 5.66, p = .001, ƞ^2^_p_=0.07, 90% CI [.02, .11]) (see Fig. 3). Planned simple contrasts showed that both snarl (F(1, 80) = 17.92, p < .001, ƞ^2^_p_ =0.18, 90% CI [.07, .30]) and puppy images (F(1, 80) = 7.16, p = .009, ƞ^2^_p_=0.08, 90% CI [.01, .19]) elicited significantly higher proportion of long responses than flower images. There was no difference between spider and flower images (p > .1, ƞ^2^_p_=0.02, 90% CI [.00, .10]). The interaction of Emotion and Duration was also significant (F(10.16, 812.64 = 3.49, p < .001, ƞ^2^_p_=0.04, 90% CI [.90, .92]).

There were no significant effects for a parallel analysis of the Weber ratio (p > .1, ƞ^2^_p_=0.02, 90% CI [.00, .05]) (see Table 3), again showing that response bias but not sensitivity was moderated by the emotional salience of the stimuli.

### Association Between Temporal Bisection, Weber Ratio and AQ

For both the temporal bisection points and Weber ratio scores, each emotional image was subtracted from the neutral image to give an index of relative overestimation and of temporal sensitivity, respectively, for each emotional image. This was done for both the face and wildlife images, resulting in six correlations (three temporal bisection point; three Weber ratio) for each category of image. There was no evidence of a substantive or significant correlation with AQ for any measure (Pearson’s r range: −0.16 to 0.08).

## Study 2: Temporal Bisection of Arousing Face and Wildlife Images in Individuals with ASD and a Matched Comparison Group

### Method

#### Participants

Twenty-four participants with a diagnosis of ASD (3 female) and 26 matched comparison participants (6 female) without a history of psychiatric or neurological disorder or illness took part. Four participants in the ASD group were excluded from further analysis as their behavioural responses in both versions of the task did not conform to a sigmoid curve suggesting that they did not discriminate between longer and shorter durations. The remaining 20 participants with ASD and all 26 matched comparison participants produced valid experimental data in at least one condition (see Results section for further details). They were recruited from a database of participants at City University, London. Participants in the clinical group were diagnosed by local health authorities and a review of medical records confirmed that all met the DSM-IV (American Psychiatric Association, 2000) criteria for ASD that were applicable at the time of their diagnosis. None of the clinical group had a history of co-morbid psychiatric disorders and none were taking prescribed medication. An additional assessment using the Autism Diagnostic Observation Schedule (ADOS: Lord et al. 1989) was possible for 18 of the participants. Fourteen met cut-off for ASD on the Communication sub-score (*M* = 2.7, range 0–5), 19 on the Reciprocal Social Interaction sub-score (*M* = 6.8, range 3–12), and 16 on the total diagnostic algorithm (*M* = 10.0, range 5–17). As clinical records confirmed the validity of the ASD diagnosis for all participants, and because excluding the participants with sub-threshold ADOS scores did not alter the pattern of significant results, all individuals were retained in the study. Groups were closely matched in terms of chronological age and IQ (Wechsler Adult Intelligence Scale III; Wechsler 1999), and the distribution of males and females was not significantly different (p = .5). In contrast, AQ scores differed significantly (see Table 4). AQ scores ranged from 22 to 45 in the ASD group, and from 4 to 23 in the comparison group. Critically, no participant in the comparison group had an AQ score over the cut-off of 32. All participants gave informed consent to take part in the study, which was approved by the Senate Ethical Committee of City University, London.

**Table 4:** Summary of Age, IQ and Autism Spectrum Quotient characteristics of the ASD and Comparison Groups in Study 2

Measure	ASD (*n* = 20: M = 17; F = 3)		Comparison (*n* = 26: M = 20; F = 6)		*p*
	*M*	*SD*	*M*	*SD*	
Age (years; months)	45;4	12.6	44;0	11.6	*ns*
Verbal IQ^a^	114.4	14.5	109.1	14.7	*ns*
Performance IQ^a^	111.0	17.8	105.6	14.3	*ns*
Full-Scale IQ^a^	114.6	16.6	108.1	15.3	*ns*
AQ^b^	35.8	14.3	14.3	5.8	< *0.001*

*M* male, *F* female

^a^Wechsler Adult Intelligence Scale III^UK^ (Wechsler 1999)

^b^Autism Spectrum Quotient (Baron-Cohen et al. 2001)

### Materials and Procedure

The materials and procedures were identical to Study 1 except that the valence and arousal ratings were not taken as Study 1 had already confirmed that the stimuli varied, as intended, on the valence and arousal dimensions. Another minor difference in methodology was that responses in Study 2 were given through the ‘1’ (re-labelled ‘S’) and ‘2’ (re-labelled ‘L’) keys (rather than ‘N’ and ‘M’) of the number-pad of the keyboard. The IQ and ADOS assessments were either already on file or taken specifically for the study but at a different time point to the experimental assessment.

## Results

For the ASD group, one participant’s face data were not collected and one participant did not produce a best-fit sigmoid-shaped curve (therefore, face n = 18). In the comparison group, one participant did not produce a best-fit sigmoid-shaped curve in the face condition (therefore, face n = 25)^1^.

### Temporal Bisection: Faces

**Fig. 4:**
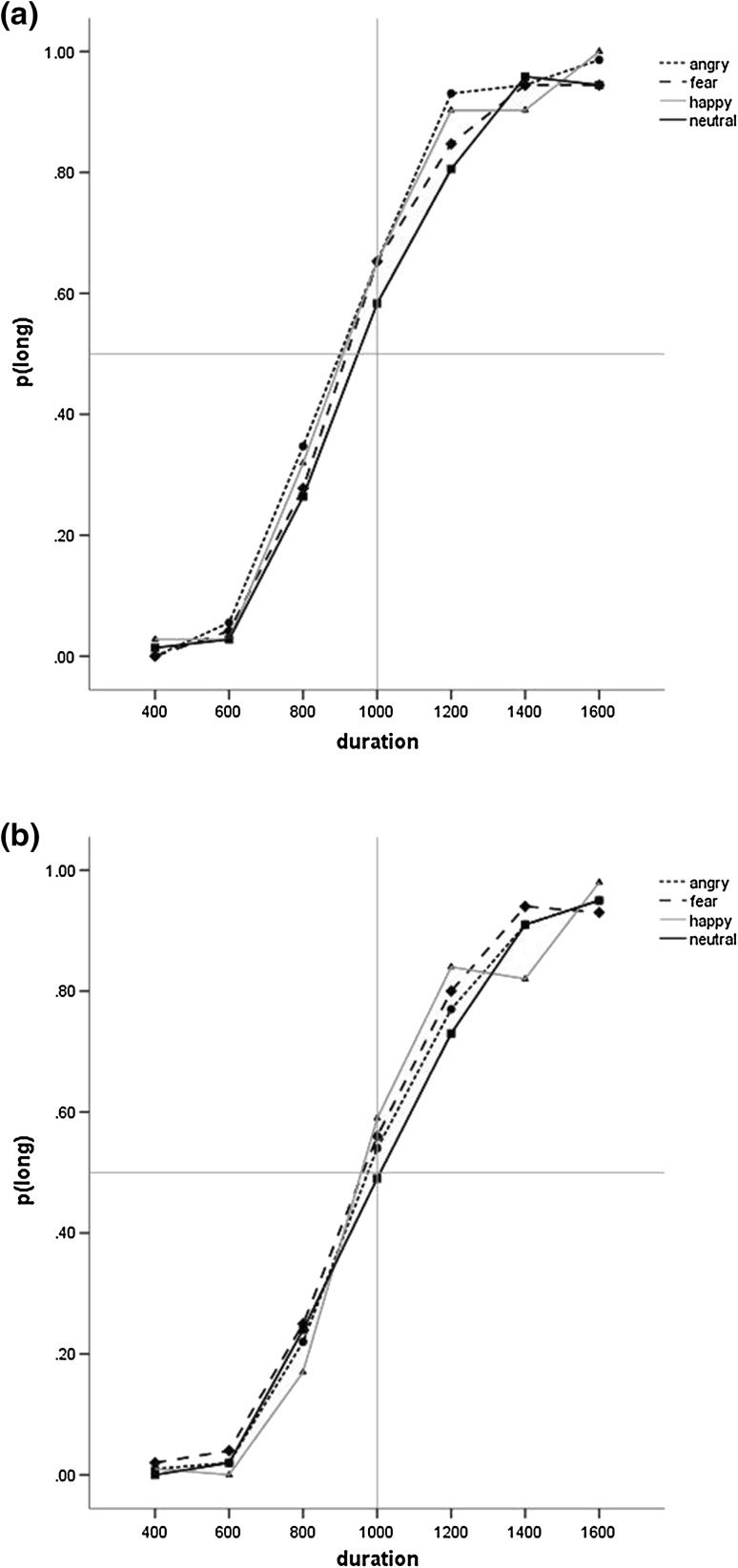
Proportion of long responses for the four facial expressions for the **a** ASD group and the **b** comparison group in Study 2. For clarity, *error bars* are omitted

A 4(Emotion) × 7(Duration) × 2(Group) ANOVA of p(long) showed a main effect of Duration (F(2.70, 110.66) = 421.95, p < .001, ƞ^2^_p_=0.91, 90% CI [.89, .93]) and a non-significant main effect of Emotion (F(3, 123) = 2.25, p = .09, ƞ^2^_p_=0.05, 90% CI [.00, .11]) (see Fig. 4). However, planned simple contrasts indicated that angry (F(1, 41) = 5.45, p = .03, ƞ^2^_p_=0.12, 90% CI [.01, .27]) and fearful (F(1, 41) = 4.25, p = .046, ƞ^2^_p_=0.09, 90% CI [.001, .25]) faces produced a significantly higher proportion of long responses than neutral faces, with the difference for happy faces being on the statistical threshold (F(1, 41) = 4.07, p = .050, ƞ^2^_p_=0.09, 90% CI [.00, .24]). Neither the main effect of group (p > .1, ƞ^2^_p_=0.05, 90% CI [.00, .19]) nor any interactions (all ps > 0.1, ƞ^2^_p_ range 0.01–0.04) were significant.

Analysis of the Weber ratio produced no significant differences (all p > .6, ƞ^2^_p_ range 0.004–0.01) (see Table 5).

**Table 5:** Average bisection points and Weber ratio for all stimulus categories for the face and wildlife images for the ASD and Comparison group in Study 2

	ASD				Comparison			
	*Bisection point*		*Weber ratio*		*Bisection point*		*Weber ratio*	
	*M*	*SD*	*M*	*SD*	*M*	*SD*	*M*	*SD*
*Face*								
Angry	906.03	148.68	0.085	0.09	1013.17	165.28	0.113	0.09
Fear	959.07	169.29	0.101	0.08	990.92	186.25	0.096	0.09
Happy	923.39	150.71	0.098	0.08	1012.50	153.34	0.103	0.06
Neutral	969.15	146.21	0.114	0.07	1027.86	178.83	0.112	0.07
*Wildlife*								
Snarl	965.39	178.50	0.119	0.08	960.05	160.85	0.117	0.08
Spider	1020.65	164.60	0.090	0.07	986.62	155.21	0.100	0.06
Puppy	976.37	187.02	0.100	0.07	974.36	151.30	0.083	0.05
Flower	1063.93	202.92	0.093	0.06	1007.10	204.51	0.105	0.08

### Temporal Bisection: Wildlife

A 4(Emotion) × 7(Duration) × 2(Group) ANOVA of p(long) showed a main effect of Duration (F(2.60, 114.18) = 505.59, p < .001, ƞ^2^_p_=0.92, 90% CI [.90, .93]) and of Emotion (F(3, 132) = 6.85, p = .001, ƞ^2^_p_=0.14, 90% CI [.05, .21]) (see Fig. 4). Planned simple contrasts showed that both snarl (F(1, 44) = 16.23, p < .001, ƞ^2^_p_=0.27, 90% CI [.10, .42]) and puppy (F(1, 44) = 10.61, p = .002, ƞ^2^_p_=0.19, 90% CI [.05, .35]) images elicited significantly higher proportion of long responses than flower images. However, there was no significant difference between spider and flower images (p > .09, ƞ^2^_p_=0.06, 90% CI [.00, .20]). Neither the main effect of Group (p > .5, ƞ^2^_p_=0.006, 90% CI [.00, .09]) nor any interactions (all ps > 0.1, ƞ^2^_p_ range 0.003–0.04) were significant.

**Fig. 5:**
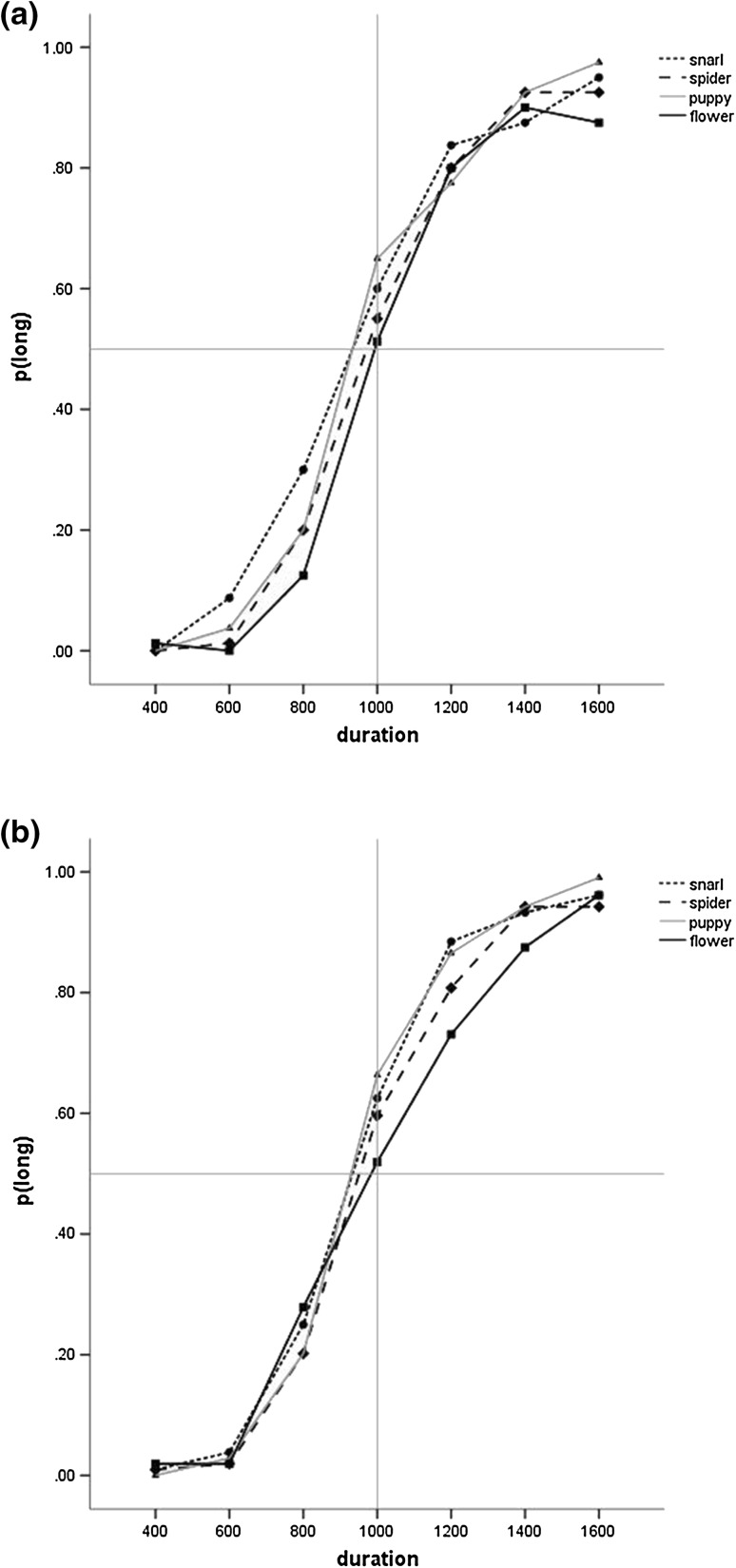
Proportion of long responses for the four wildlife images for the **a** ASD group and the **b** comparison group in Study 2. For clarity, *error bars* are omitted

There were no significant effects for the parallel analysis of the Weber ratio (all ps > 0.1, ƞ^2^_p_ range 0.000–0.04) (see Table 5; Fig. 5).

### Association Between Temporal Bisection, Weber Ratio and AQ

As with Study 1, indices of relative overestimation and temporal sensitivity for each emotional image were calculated, with correlations for each group performed separately. For the ASD group, a significant negative correlation was found between the change in temporal sensitivity in the fearful face condition (compared to neutral) and the AQ (r=−0.55; p = .019). This would suggest *better* temporal sensitivity for fearful faces is associated with higher self-reported autistic traits. However, this finding was not significant using a Bonferroni corrected p value (three Weber ratio correlations for face stimuli) of 0.017. Further, a post-hoc independent samples t-test established a non-significant difference in Weber ratio between groups in the fear condition, for both absolute Weber ratio scores and for the Weber score relative to neutral (p > .8). No other correlations, for either group, were significant (Pearson’s r range −0.31 to 0.36).

## Discussion

Using a paradigm novel to ASD research, this study investigated implicit responsiveness to emotionally-charged faces and wildlife images in ASD. We found that adults with ASD and without intellectual impairment did not differ significantly from a matched comparison group and showed a similar tendency to overestimate intervals in which an emotional image was presented. Parallel measurement of effect size indicated a negligible effect of group membership on the estimation of wildlife images and a small-to-medium effect for the face images. Consistent with this, the performance of adults in the general population did not correlate with self-reported autistic traits. Taken together, these results do not suggest a meaningful difference between ASD and comparison populations in the implicit behavioural reaction to arousing stimuli, whether emotional faces or hedonically matched wildlife images. Before we consider the implications of this finding in relation to the emotion-processing literature in ASD, we will briefly consider the implications of our observations on temporal processing in ASD.

### Intact Temporal Perception in ASD

Although the primary focus of this study was on the implicit processing of emotion, the temporal bisection task is a direct and explicit measure of interval timing. Therefore, the findings are relevant to the hypothesis that ASD is underpinned by a fundamental disturbance to an internal clock mechanism, which is an argument that has gained ground in recent years. However, results in relation to this hypothesis are equivocal, with evidence of intact (Jones et al. 2009; Gil et al. 2012; Mostofsky et al. 2000; Wallace and Happé 2008) and impaired (Allman et al. 2011; Brenner et al. 2015; Brodeur et al. 2014; Falter et al. 2012; Karaminis et al. 2016; Kargas et al. 2015; Maister and Plaisted-Grant 2011; Martin et al. 2010; Szelag et al. 2004) temporal processing in the millisecond and seconds range. The current study does not support a fundamental timing disturbance in adults with ASD who have no intellectual impairments. The varied results could relate to both the heterogeneity of the tasks used and the population being tested (see Jones and Jahanshahi 2014). A key consideration is that interval timing cannot be dissociated from a range of ancillary cognitive processes, including attention, working memory, decision making and motor execution, which are all additional sources of variance. Maister and Plaisted-Grant (2011) argued that attentional variability explained the poor performance of their ASD group on a measure of time reproduction of 0.5 s intervals, while insufficient episodic processes explained poor performance on 45 s intervals. Further, Brenner et al. (2015) found evidence that consistency of responding on a measure of time reproduction was associated with auditory working memory ability in children and adolescents with ASD. Stewart et al. (2015) recently found that autistic traits in the general population correlated positively with performance on a duration discrimination task (comparing a standard and comparison interval) when a fixed standard interval was used but not when a variable standard was used. They suggest high levels of autistic traits may be associated with an enhanced ability to form a stable and accurate perceptual representation of a repeated stimulus, a mechanism that is not limited to temporal processing. More thorough exploration of the temporal processing profile in ASD is needed, including testing across a range of tasks and durations (see Jones and Jahanshahi 2014 for discussion of this approach in a different clinical population).

To our knowledge, three previous studies have used the temporal bisection task in ASD. Two report impairment in children with ASD with a mean age of 10 years (Allman et al. 2011; Brodeur et al. 2014). However, Allman et al., did not measure IQ in the majority of their comparison group, which means that IQ matching between groups could not be established. Brodeur et al. (2014) found evidence of flatter temporal bisection slopes in children with ASD and generally low mental age, indicating less sensitivity to duration. They remark that the shallower slope reflects the findings in younger TD children (McCormack et al. 1999) and suggest a developmental delay that partly relates to less well-developed attention mechanisms. However, Gil et al. (2012) found no evidence of impairment across four temporal bisection tasks in autistic children and adolescents with no reported intellectual impairments. The most obvious difference between studies is the level of intellectual functioning of the ASD groups. However, Brodeur et al. also used pure tone stimuli, compared to a simple visual display in the Gil et al. study. Given known differences in the way that auditory and visual stimuli are timed (Wearden et al. 1998), it may be relevant to further investigate modality effects. To summarise across the current and previous studies, it can be concluded that both adults and adolescents with ASD and without intellectual impairment are relatively unimpaired on the temporal bisection task when visual stimuli are used. However, further work using children and testing across the range of intellectual ability will be important for fully understanding the profile of ability.

### Intact Sensitivity to Emotionally Arousing Images in ASD

Returning to the primary focus of this study, the data suggest that adults with ASD demonstrate an implicit responsiveness to emotional stimuli that is not meaningfully distinguishable from those without ASD. This conclusion is supported by the lack of group differences in Study 2 as well as the absence of reliable correlations between autistic traits and relevant indices of emotional responsiveness across both experiments. It is notable that the pattern of performance was observed for both emotional face stimuli and for hedonically similar wildlife stimuli. The common explanation for overestimation on the temporal bisection task is that arousal has been elevated (e.g. Droit-Volet et al. 2004). The current data therefore suggest that the psychophysiological response to simple emotional images in ASD is relatively intact. However, caution must be exercised in the absence of confirmatory physiological data. This is particularly the case as the multiple cognitive mechanisms inherent to the temporal judgement (e.g. working memory, attention, decision making) are other sources of variance that may be affected by emotional content (e.g. Droit-Volet and Gil 2009; Gibbon et al. 1984). Indeed, it is assumed that emotion also has an impact on attentional mechanisms relevant to timing, which can enhance or diminish the arousal effect (e.g.Droit-Volet et al. 2013). On the basis of the current data, we cannot rule out the possibility that superficially similar patterns of behavioural responses in ASD are mediated by a different combination of underlying processes compared to the comparison group. Systematic investigation, which varies conditions to probe all relevant processes, is necessary to shed light on this issue.

A pertinent question is how well the current data fit with other studies that have sought to characterize implicit emotional responses in ASD. Hubert et al., (2009) measured SCR during an explicit labelling task, using video clips of actors expressing happiness or anger. Supporting the current study, there was no significant difference in SCR between those with and without ASD when the emotional states were implicitly processed. Another measure of implicit response to emotion is the production of automatic facial muscle movements that mimic the emotion being portrayed. Mimicry is argued to induce an internal simulation of the emotion, leading to a physiological response and consequences for emotion recognition, empathy and emotional reciprocity (e.g. Niedenthal 2007). It has been argued that facial mimicry of emotions triggers the increased arousal driving the overestimation of facial displays of emotion (Effron et al. 2006). Evidence that overestimation of emotional faces is extinguished when participants’ facial movements are inhibited (e.g. holding a pen in the mouth) has provided support for this theory (Effron et al. 2006). The majority of studies on facial mimicry in ASD report evidence of impairment (Beall et al. 2008; Mathersul et al. 2013; McIntosh et al. 2006; Rozga et al. 2013). However, there is some suggestion of delayed development of the mimicry response, rather than absolute absence (Beall et al. 2008), and studies have shown that typical mimicry responses can occur but with a delayed onset (Mathersul et al. 2013; Oberman et al. 2007). Further, Magneé et al. (2007) found no evidence of impairment in EMG response in adults with ASD. As with the current study, the participants were engaged in a non-emotion task (sex judgement) so were not explicitly oriented to the emotional cues. If, as others suggest (e.g. Effron et al. 2006), mimicry is necessary for the overestimation of emotional faces, then the current results indicate that any differences in the production of facial mimicry in ASD are not sufficient to significantly disrupt performance on the task.

Considering some of the wider implications of preserved implicit emotion processing in ASD, the data suggest that individuals with and without ASD experience similar distortions in the perception of the temporal unfolding of emotional events. These distortions have been argued to play an important role in social interaction (see Droit-Volet et al. 2013; Droit-Volet and Gil 2009). Indeed, social interaction can be conceptualised as a series of complex, self-organising temporal dynamics between interlocutors (Fusaroli et al. 2014). For example, successful conversational interaction involves synchronising of speech rate (e.g. Schultz et al. 2016) and movement (e.g. Ramseyer and Tschacher 2011), the co-ordination of visual attention (e.g. Richardson et al. 2007), and timely turn-taking (e.g. Levinson and Torreira 2015). The extent to which this broad category of ‘social timing’ may be impaired in ASD has thus far received limited attention (see Wimpory et al. 2002). The speeding of time when presented with emotional faces has been theoretically related to the need for action readiness, with timely preparation to act leading to effective anticipation of a social partner’s subsequent behaviour (see Droit-Volet et al. 2013; Droit-Volet and Gil 2009). Knowledge of ASD predicts that this preparation to act is not utilised in a typical way, perhaps because intrinsic understanding of *how* to act is not forthcoming. Indeed, Loveland (1991, 2005) suggested that individuals with ASD are not necessarily insensitive to the emotions of others but that they experience difficulties regulating their own behaviour accordingly. Such difficulties early in life could lead to abnormalities in the explicit understanding of the expressions of others, which would be mediated by a complex set of interacting processes (e.g., language, perception, attention, memory etc.). Implicitly responding to the emotions of others, however, could remain spared.

### Limitations

Before concluding, it is important to acknowledge some possible limitations of the current study. First, the use of static stimuli dictates that replication is required with more ecologically valid stimuli, such as dynamic videos. This is particularly the case given the modest effect size found between groups for the face stimuli, and the suggestion in the literature that ecologically valid stimuli can pose greater difficulties for individuals with ASD (e.g., Gaigg 2012). It could be argued that the temporal overestimation of arousing events becomes heightened when stimuli become more complex and the emotional response is triggered in multiple channels (e.g. auditory, visual). Whether individuals with ASD would show the same ramping response predicted in a typical population would give further insight into their processing of emotion in real life situations. Similarly, the emotions used in the current study were all ‘basic’ (e.g. Ekman 1992). Using the same paradigm but with more complex emotions would establish whether there are limitations to this particular type of emotional responsiveness in ASD.

It is also important to recognise that a null effect of group in Study 2 could reflect issues relating to power or sampling, and replication of these findings is therefore necessary, particularly with more heterogeneous samples in terms of chronological age and language/intellectual ability. Similarly, although our typical population presented with a range of AQ scores that would be expected in the general population (Baron-Cohen et al. 2001), a wider spread of scores would have provided more variance for detecting an experimental effect. Despite these caveats, there is strength in our replication of findings across two studies that use two different categories of image, and in our use of a paradigm that yields replicable effects across numerous studies in the literature.

## Conclusions

Across two separate experiments, we established that ASD and autistic traits appear to be associated with a typical implicit behavioural response to simple emotional images, both face and wildlife. This is the first time the paradigm has been used with an ASD population and demonstrates the value of testing implicit responses, which circumvent issues with strategy use or uncontrolled cognitive or perceptual variables. The majority of research into ASD is focused on highlighting and delineating differences from individuals not on the spectrum. The current data provide evidence of a circumstance in which individuals with and without ASD appear to have a shared perceptual experience.

## Footnotes

1 Due to the modest sample size in this Study, it is questionable whether parametric or non-parametric inferential statistics are most appropriate. Since both approaches yield the same pattern of results, and for consistency with Study 1, parametric statistics are reported throughout the results.
